# Prognostic and Clinicopathological Significance of CD74 Expression in Pancreatic Ductal Adenocarcinoma: A Systematic Review and Meta-Analysis

**DOI:** 10.3390/ijms27198488

**Published:** 2026-09-23

**Authors:** Ioan Alexandru Vacariu, Silvia Mihaela Ilie, Cristin Constantin Vere, Adina Turcu-Stiolica, Eleni Manthopoulou, Mihai Radu Pahomeanu, Eugen Tcaciuc, Sebastian Constantin Toma, Bogdan Silviu Ungureanu

**Affiliations:** 1 Doctoral School, University of Medicine and Pharmacy of Craiova, 200349 Craiova, Romania; vacariu_alexandru99@yahoo.com; 2 Department of Medical Oncology, Georges Francois Leclerc Cancer Center, 21079 Dijon, France; silie@cgfl.fr; 3 Cancer Biology Transfer Platform, Georges Francois Leclerc Cancer Center, 21079 Dijon, France; 4 INSERM UMR 1231, Center for Translational and Molecular Medicine (CTM), University of Burgundy Europe, 21079 Dijon, France; 5 Department of Gastroenterology, Faculty of Medicine, University of Medicine and Pharmacy of Craiova, 200349 Craiova, Romania; bogdan.ungureanu@umfcv.ro; 6 Biostatistics Department, Faculty of Pharmacy, University of Medicine and Pharmacy of Craiova, 200349 Craiova, Romania; 7 Health Economics and Outcomes Research Department, Faculty of Medicine, “Iuliu Haţieganu” University of Medicine and Pharmacy Cluj-Napoca, Victor Babes Street No. 8, 400012 Cluj-Napoca, Romania; 8 Gastroenterology Department, General Hospital of Nea Ionia “Konstantopouleio-Patision”, 3–5 Theodorou Konstantopoulou Street, Nea Ionia, 14233 Athens, Greece; e-manthopoulou@hotmail.com; 9 Internal Medicine and Gastroenterology Department, Faculty of Medicine, Carol Davila University of Medicine and Pharmacy, 050474 Bucharest, Romania; mihairadu.pahomeanu@gmail.com; 10 Internal Medicine and Gastroenterology Department, University Emergency Hospital of Bucharest, 050098 Bucharest, Romania; 11 Department of Gastroenterology, Nicolae Testemitanu State University of Medicine and Pharmacy, MD-2004 Chisinau, Moldova; eugentcaciuc@yahoo.com; 12 General Surgery Department, Dr. Constantin Andreoiu, Ploiesti County Emergency Hospital, 100097 Ploiesti, Romania; tosecon@hotmail.com

**Keywords:** CD74, pancreatic ductal adenocarcinoma, PDAC, perineural invasion, lymph node metastasis, prognosis, meta-analysis

## Abstract

Pancreatic ductal adenocarcinoma (PDAC) remains one of the most lethal solid malignancies, and reliable prognostic biomarkers are urgently needed to guide risk stratification and treatment. CD74 has been implicated in tumor progression, invasion, and pro-inflammatory signaling in various cancers. This systematic review and meta-analysis aimed to evaluate the association between CD74 expression and key clinicopathological features and survival outcomes in patients with PDAC. A systematic literature search was conducted in PubMed, Web of Science, and Scopus to identify eligible studies reporting on CD74 expression in PDAC tissues (PROSPERO: CRD420261402709). Studies were selected following the PRISMA 2020 guidelines. The methodological quality of the included studies was assessed using the QUIPS tool. Pooled odds ratios (ORs) with 95% confidence intervals (CIs) were calculated for the associations between CD74 expression and perineural invasion (PNI), lymphatic invasion, lymph node metastasis (pN), vascular permeation, pTNM stage, and overall survival. Statistical analyses were performed using Review Manager v5.4. DerSimonian–Laird random-effects models were applied as sensitivity analysis for every outcome, with Mantel–Haenszel fixed-effects models as the primary analysis according to the level of heterogeneity, which was quantified using the I^2^ statistic. A total of 83 records were initially identified, of which five studies met the inclusion criteria and were included in the quantitative synthesis. CD74-positive expression was significantly associated with an increased risk of perineural invasion (four cohorts, 351 patients; OR = 3.69; 95% CI: 2.27–5.99; *p* < 0.00001; I^2^ = 0%), lymphatic invasion (four cohorts, 351 patients; OR = 2.05; 95% CI: 1.21–3.46; *p* = 0.007; I^2^ = 0%), and lymph node metastasis (four cohorts, 462 patients; OR = 1.94; 95% CI: 1.27–2.96; *p* = 0.002; I^2^ = 0%). A non-significant trend toward increased vascular permeation (three cohorts, 181 patients; OR = 1.99; 95% CI: 0.84–4.68; *p* = 0.12; I^2^ = 0%) and advanced pTNM stage (three cohorts, 284 patients; OR = 1.62; 95% CI: 0.93–2.82; *p* = 0.09; I^2^ = 0%) was also observed. Importantly, CD74 positivity was significantly associated with worse overall survival (three cohorts, 293 patients; HR = 2.04, 95% CI: 1.45–2.88; *p* < 0.00001; I^2^ = 0%). This analysis was structured into two subgroups: HR = 2.21 (95% CI: 1.50–3.25) in the two cohorts contributing multivariable-adjusted, immunohistochemistry-based estimates, and HR = 1.52 (95% CI: 0.72–3.21) in the single cohort contributing a univariable estimate reconstructed from transcript-level data, with no significant difference between the subgroups (*p* = 0.38). Because the estimated between-study variance was zero for every outcome, random-effects estimates were almost identical to the fixed-effects estimates throughout. As the pooled survival estimate combines adjusted with unadjusted estimates, it describes a prognostic association rather than established independent prognostic value. This meta-analysis demonstrates that elevated CD74 expression in PDAC is significantly associated with perineural invasion, lymphatic invasion, lymph node metastasis, and poorer overall survival, supporting an association with an adverse prognostic. Because the evidence base comprises few retrospective cohorts that used different, non-standardized thresholds to define CD74 positivity and different definitions of several outcomes, these findings should be regarded as hypothesis-generating: they identify CD74 as a candidate for prospective validation and mechanistic investigation rather than as an established biomarker for risk stratification or therapeutic decision-making. Further large-scale prospective studies are warranted to validate these results and explore the clinical utility of CD74-targeted strategies.

## 1. Introduction

Pancreatic ductal adenocarcinoma (PDAC) remains one of the most lethal solid malignancies, with a wide search for reliable prognostic biomarkers to guide risk stratification and treatment [1]. Perineural invasion (PNI) is increasingly recognized as a critical determinant of PDAC aggressiveness, and it occurs in nearly all cases, far exceeding its prevalence in other solid tumors and reflecting the pancreas’ rich surrounding nerve plexus. PNI is associated with poor prognosis, tumor recurrence, and pain, and invaded nerves display widespread plasticity with a pattern consistent with neuronal injury, a remodeling process termed neuroplasticity that PDAC cells can actively induce [2].

CD74, also known as the MHC class II invariant chain (Ii), is a type II transmembrane glycoprotein that performs several distinct functions. In the endoplasmic reticulum it tri-merises and associates with newly synthesized MHC class II αβ dimers, occupying the peptide-binding groove through its CLIP segment, preventing premature peptide loading and routing the complex to the endolysosomal compartment, where sequential proteolysis and HLA-DM-mediated CLIP exchange complete class II maturation. A minor fraction of CD74 reaches the plasma membrane, where it acts as an accessory signaling receptor governing B-cell and dendritic-cell maturation, motility and survival. CD74 is constitutively expressed by antigen-presenting cells and can be induced in epithelial, endothelial and stromal cells under inflammatory conditions, a point that is important when tissue-level staining in tumors is interpreted. Moreover, it has emerged as a key player in cancer biology by serving as the cell-surface receptor for macrophage migration inhibitory factor (MIF), thereby activating downstream signaling pathways such as PI3K/AKT, ERK, and NF-κB that promote tumor cell survival, proliferation, and immune evasion [3].

Several pan-cancer analyses have reported elevated CD74 expression in hematologic malignancies, gastric and colorectal cancers, melanoma, glioma, and non-small-cell lung cancer compared to normal tissue [4,5]. Its clinical meaning is context-dependent: in many tumor types, high CD74 expression is associated with improved patient prognosis, likely because it correlates positively with infiltrating B cells, CD4 and CD8 T cells, neutrophils, macrophages, and dendritic cells, whereas in other settings CD74 promotes tumor proliferation and is associated with worse survival.

In PDAC, CD74 is overexpressed and behaves as an oncogenic driver. It has been shown that CD74 signaling drives the expression of cytokines S100A8 and S100A9 through activation of the TRAF6–NF-κB pathway, thus promoting a pro-inflammatory cytokine release from fibroblasts [6]. On the other hand, CD74 activates the PI3K/AKT signaling cascade, leading to induction of the early growth response-1 (EGR-1) transcription factor. EGR-1 transactivates the glial cell line-derived neurotrophic factor (GDNF) promoter, resulting in increased GDNF biosynthesis and secretion proportional to CD74 expression [6]. Secreted GDNF acts in a paracrine manner on intratumoral autonomic and sensory nerves to promote axonogenesis and neural hypertrophy (neuroplasticity), while CD74 concurrently augments the migratory and invasive phenotype of the carcinoma cells.

A refined overview of CD74 expression in PDAC is warranted due to growing evidence of its involvement in tumor progression, immune modulation, and patient prognosis. Reported associations between CD74 expression and clinical endpoints are inconsistent in direction, reflecting differences in detection methods and prognosis. Thus, we proposed a meta-analysis of all available studies that focused on the immunohistochemical expression of CD74 in PDAC to clarify its prognostic significance and guide future biomarker-driven research.

## 2. Materials and Methods

### 2.1. Study Design and Protocol Registration

This systematic review and meta-analysis were conducted and reported in accordance with the Preferred Reporting Items for Systematic Reviews and Meta-Analyses (PRISMA) 2020 guidelines (Supplementary File S1) [7]. The protocol was registered into PROSPERO database (https://www.crd.york.ac.uk/PROSPERO/view/CRD420261402709, accessed on 15 August 2026).

A systematic literature search was performed from inception to 20 May 2026, to identify all relevant sources from PubMed, Web of Science, and Scopus. The full search strategy for the three databases is presented in the Supplementary File S2.

Studies were considered eligible for inclusion if they met the following criteria: (1) original observational studies (cohort or case–control) involving patients with histologically confirmed PDAC; (2) evaluation of CD74 expression in human tumor tissues, typically by immunohistochemistry; (3) reporting of the association between CD74 expression status (positive vs. negative, or high vs. low) and at least one clinicopathological feature (perineural invasion, lymphatic invasion, lymph node metastasis, vascular permeation, pTNM stage) or survival outcome; and (4) provision of sufficient data to calculate odds ratios (ORs) with 95% confidence intervals (CIs) or hazard ratios (HRs) with corresponding standard errors (SEs).

Exclusion criteria included: (1) studies with inappropriate design; (2) studies restricted to cell line experiments without human tissue analysis; (3) studies focused on cancer types other than PDAC; (4) reviews, editorials, conference abstracts, case reports, and letters; and (5) studies lacking extractable data despite contacting the corresponding authors.

### 2.2. Data Extraction, Quality Assessment and Publication Bias

All retrieved records were exported to Rayyan management software [8], and duplicates were removed. Two independent reviewers screened the titles and abstracts of all unique records against the predefined eligibility criteria (A.V. and A.T.-S.). Articles deemed potentially eligible underwent full-text review. Any disagreements between reviewers were resolved through discussion or, when necessary, consultation with a third reviewer (B.S.U.).

Two reviewers independently extracted data from the included studies using a standardized data extraction form (I.A.V. and A.T.-S.). The following information was collected: first author’s name, year of publication, country, study design, sample size, patient demographics, CD74 detection method and scoring criteria, cut-off values for CD74 positivity, clinicopathological parameters (perineural invasion, lymphatic invasion, lymph node metastasis, vascular permeation, pTNM stage), and survival data (HR with 95% CI or log[OR] with SE). When only the HR and 95% CI were available, the SE of the log(HR) was derived using the formula: SE[log(HR)] = (ln(upper CI) − ln(lower CI))/3.92. For studies that did not report HRs directly, HRs and their 95% CIs were reconstructed from digitized Kaplan–Meier survival curves using the methods described by Tierney et al. Survival curve data were extracted using a graphical digitization tool (WebPlot Digitizer, Version 4.7, Austin, TX, USA) [9]. Discrepancies were resolved by consensus or through arbitration by a third reviewer (B.S.U.).

For every cohort contributing to the survival synthesis we additionally recorded (i) whether the hazard ratio was reported directly by the original authors, reconstructed from a digitized Kaplan–Meier curve, or derived from an external gene-expression database, and (ii) whether the estimate was univariable or adjusted in a multivariable model, together with the variables entering that model. Where a multivariable hazard ratio was available in the primary publication it was preferred over an unadjusted estimate.

Risk of bias in prognostic studies was assessed using the Quality in Prognosis Studies (QUIPS) tool, being specifically designed for prognostic factor research and to capture sources of bias that are especially relevant to biomarker studies [10]. The tool includes cohort selection, completeness of follow-up, measurement of the prognostic factor, outcome ascertainment, control of confounding, and the appropriateness of statistical analysis and reporting. Each eligible study was evaluated across the six QUIPS domains—study participation, study attrition, prognostic factor measurement, outcome measurement, study confounding, and statistical analysis and reporting—and each domain was judged as low, moderate, or high risk of bias. Studies that did not include a true time-to-event prognostic design were not considered fully assessable within the QUIPS framework. The domain-level judgment for each individual study, together with a graphical summary, is presented in Supplementary File S3, underlying which domains—particularly prognostic factor measurement, study confounding, and statistical analysis and reporting—drove the overall risk-of-bias assessment. Two reviewers independently assessed each study (I.A.V. and A.T.-S.), with discrepancies resolved by consensus or by arbitration by a third reviewer (B.S.U.).

Certainty in the body of evidence was rated at outcome level using the GRADE approach adapted to prognostic factor research. Under this adaptation, evidence assembled from adequately conducted prognostic factor studies begins at high certainty rather than at the low starting point applied to observational studies of interventions, because a well-designed prognostic study is the appropriate design for a prognostic question. Certainty was then downgraded by one or two levels in each of five domains (study limitations, inconsistency, indirectness, imprecision and publication bias) drawing on the study-by-study QUIPS judgements, and could be upgraded for a large effect, a dose–response gradient or for plausible residual confounding acting against the observed association. Two reviewers rated each domain independently (I.A.V. and A.T.-S.), with disagreements resolved by consensus or by arbitration by a third reviewer (B.S.U.).

### 2.3. Statistical Analysis

All statistical analyses were performed using Review Manager (RevMan, version 5.4.1, Cochrane Collaboration, 2020, Copenhagen, Denmark). For dichotomous clinicopathological outcomes, pooled odds ratios (ORs) with 95% confidence intervals (CIs) were calculated using the Mantel–Haenszel (M–H) method. For survival outcomes, pooled effect estimates were obtained using the generic inverse variance (IV) method based on log[HR] and corresponding standard errors (SEs).

Statistical heterogeneity among studies was assessed using Cochran’s Q test (Chi^2^ statistic) and quantified with the I^2^ statistic. An I^2^ value of < 25% was considered low heterogeneity, moderate for 25–50%, substantial for 50–75%, and considerable heterogeneity for >75%. A fixed-effects model was applied when no significant heterogeneity was detected (I^2^ ≤ 50%, *p* > 0.10); otherwise, a random-effects (DerSimonian–Laird) model was used. Cochran’s Q and I^2^ have very low power when only three or four cohorts contribute to an outcome, so that an observed I^2^ of 0% cannot be taken as evidence of homogeneity. A subgroup analyses by cut-off threshold or geographic origin were planned but not feasible due to the small number of studies.

The overall effect was tested using the Z-test, with a two-sided *p*-value of < 0.05 considered statistically significant. Results are presented as forest plots displaying individual and pooled effect estimates with 95% CIs.

To evaluate the robustness of the pooled estimates and identify potentially influential studies, leave-one-out sensitivity analyses were performed by sequentially excluding each study and recalculating the pooled effect size. This approach was particularly important for outcomes exhibiting substantial heterogeneity.

Due to the limited number of studies included per outcome (<10), formal assessment of publication bias using funnel plot inspection or Egger’s regression test was not performed, in accordance with the recommendations of the *Cochrane Handbook for Systematic Reviews of Interventions*, which states that such tests have insufficient statistical power when fewer than 10 studies are available. The absence of formal testing must not be read as evidence that publication bias or small-study effects are absent. Biomarker studies of this size are particularly susceptible to selective publication of positive associations; therefore, the pooled estimates may be biased away from the null.

A summary of the statistical methods, effect measures and indices used in the quantitative synthesis is provided in Table 1.

### 2.4. Search Results

A systematic literature search across three electronic databases yielded a total of 83 records, comprising 30 from PubMed, 29 from Web of Science, and 24 from Scopus. After removing 42 duplicate records, 41 unique articles were screened based on title and abstract. Of these, 34 articles were excluded for the following reasons: 15 had an inappropriate study design; 10 were limited to cell line experiments; 5 investigated other types of cancer, and 4 were review articles. The remaining 7 articles underwent full-text assessment for eligibility, after which 2 articles were further excluded due to the absence of extractable data. Ultimately, 4 articles [11,12,13,14] (1 article included 2 different cohorts [14]) met all inclusion criteria and were incorporated into the final meta-analysis. The study by Hong et al. [6] was included in the systematic review due to the contributing data to the overall survival analysis (transcript-level prognostic analyses of human tumor tissue). The complete study selection process is illustrated in Figure 1, following the PRISMA guidelines.

## 3. Results

### 3.1. Study Characteristics

The systematic search identified five eligible studies (Hong 2023 [6], Koide 2006 [11], Nagata 2009 [12], Zhang 2014 [13], and Zhang 2021 [14] with two different cohorts) that quantitatively reported associations between CD74 expression and clinicopathological or prognostic endpoints in human pancreatic neoplasms, and that provided data amenable to pooling. Across the six cohorts, 687 patients contributed data to at least one analysis (Hong 2023 [6], 179 TCGA tumors; Koide 2006 [11], n = 67; Nagata 2009 [12], n = 68; Zhang 2014 [13], n = 46; Zhang 2021-R1 [14], n = 157; Zhang 2021-R2 [14], n = 170). The number of cohorts and patients contributing to each endpoint was as follows: perineural invasion, four cohorts and 351 patients; lymphatic invasion, four cohorts and 351 patients; lymph node metastasis, four cohorts and 462 patients; vascular permeation, three cohorts and 181 patients; pTNM stage, three cohorts and 284 patients; and overall survival, three cohorts and 293 patients. CD74 status was determined immunohistochemically in five of the six cohorts and, in the study by Hong et al. [6], from TCGA transcript-level data accessed through GEPIA2. Patients were dichotomised into CD74-positive or high-CD74+ and CD74-negative or low-CD74− groups using the threshold defined by the original investigators. These thresholds differ substantially (≥10%, ≥25% and ≥70% stained cells, and the GEPIA2 median expression cut-off) and are not necessarily biologically interchangeable; pooled results therefore refer to study-defined high- or positive-CD74 expression throughout rather than to any standardized definition of positivity. The definitions of several outcomes also differ between cohorts: Koide 2006 [11] graded perineural, lymphatic and vascular invasion from zero to three and dichotomised them as grades 2–3 versus 0–1 rather than as present versus absent, and the contrast used for advanced pTNM stage was stages III+IV versus I+II in Nagata 2009 [12], stage III versus I+II in Zhang 2021-R2 [14] and stage II versus stage I in Zhang 2014 [13], which recruited only stages I–II patients. The endpoints reported with sufficient frequency for meta-analysis were lymphatic invasion, perineural invasion, vascular permeation, lymph node metastasis (pN status), pTNM stage, and overall survival. Not every study contributed data to every endpoint; the number of contributing studies and the patient totals per analysis are summarized in Table 2.

Zhang 2021 [14] contributed two institutional cohorts, Renji cohort 1 (R1, recruited from January 2002 to June 2013) and Renji cohort 2 (R2, recruited from July 2013 to December 2016). The recruitment windows of R1 and R2 do not overlap, so no patient can appear in both. Zhang 2014 [13] reported a separate series of resectable stages I–II patients recruited between January 2008 and December 2009 at the same institution, a period that fell within the R1 recruitment window, so overlapping patients between those two datasets were possible and cannot be excluded from the published data, in which individual patient identifiers were not available. We therefore never combined Zhang 2014 [13] and Zhang 2021-R1 [14] within the same analysis: every endpoint draws on at most one of them, and the possibility of partial overlap is acknowledged in the Limitations section.

Risk of bias across the included CD74 prognostic studies was generally low to moderate (Supplementary File S3). The strongest study was from Zhang 2021 [14], which included larger resected PDAC cohorts and internal validation, but it remained vulnerable to bias from its retrospective single-center design, exclusion-based cohort assembly, study-specific IHC cut-off, and incomplete control of confounding. Earlier clinical studies, particularly Nagata 2009 [12] and Zhang 2014 [13], were judged at moderate risk of bias overall because they were small retrospective surgical series with selected patient populations, limited follow-up reporting, non-standardized or data-influenced CD74 thresholds, and sparse multivariable adjustment. The prognostic component of the study by Hong 2023 was also judged at moderate risk of bias, as it relied on secondary TCGA/GEPIA mRNA survival analyses without adequate multivariable adjustment or directly reported effect estimates suitable for quantitative synthesis. Overall, the evidence suggesting adverse prognostic significance of CD74 in PDAC should therefore be interpreted cautiously, particularly given the predominance of retrospective single-center studies.

### 3.2. Association of CD74 with Clinicopathological Features

The primary estimates reported below are Mantel–Haenszel fixed-effects estimates. Because the between-study variance was estimated as zero for every outcome (τ^2^ = 0.00; I^2^ = 0%), the corresponding DerSimonian–Laird random-effects estimates were almost identical; they are given in parentheses after each primary result, and the full random-effects output is summarized in Table 3. No single cohort, when omitted, altered the direction or the statistical significance of any pooled estimate.

#### 3.2.1. Perineural Invasion

A fixed-effects meta-analysis of four studies (Koide 2006 [11], Nagata 2009 [12], Zhang 2014 [13], and Zhang 2021-R2 [14]) including 208 CD74+ and 143 CD74- patients (351 in total) demonstrated that CD74 positivity was significantly associated with an increased likelihood of perineural invasion. The pooled odds ratio of perineural invasion was more than three-fold higher in CD74+ tumors (OR of 3.72, 95% CI 2.30–6.02; Z = 5.35, *p* < 0.00001). The individual study estimates were concordant in direction and magnitude (Koide 2006 [11], OR of 4.93; Nagata 2009 [12], OR of 4.43; Zhang 2014 [13], OR of 7.00; Zhang 2021-R2 [14], OR of 2.88), with no statistical heterogeneity (τ^2^ = 0.00; Chi^2^ = 1.62, df = 3, *p* = 0.66; I^2^ = 0%). The random-effects meta-analysis demonstrated the same association (OR of 3.69, 95% CI 2.27–5.99; Z = 5.28, *p* < 0.00001), as shown in Figure 2. Koide 2006 [11] contributed a comparison of perineural invasion grades 2–3 versus 0–1 rather than present versus absent, so the pooled estimate combines two slightly different contrasts. No overlapping patients were included in the current meta-analysis between the two reports from Zhang 2014 [13] and Zhang 2021 [14].

#### 3.2.2. Lymphatic Invasion

A fixed-effects meta-analysis of four studies (Koide 2006 [11], Nagata 2009 [12], Zhang 2014 [13], and Zhang 2021-R2 [14]) comprising 207 CD74+ and 144 CD74- patients evaluated the relationship between CD74 expression and lymphatic invasion. The pooled OR was 2.05 (95% CI: 1.22–3.46; Z = 2.70, *p* = 0.007), indicating a significantly increased lymphatic invasion in CD74+ patients. The random-effects meta-analysis demonstrated the same association (OR of 2.05, 95% CI 1.21–3.46; Z = 2.68, *p* = 0.007), as shown in Figure 3. No overlapping patients were included in the current meta-analysis between the two reports from Zhang 2014 [13] and Zhang 2021 [14].

#### 3.2.3. Lymph Node Metastasis (pN Status)

A fixed-effects meta-analysis of four studies (Koide 2006 [11], Nagata 2009 [12], Zhang 2021-R1 [14], and Zhang 2021-R2 [14]; 273 CD74+ and 189 CD74- patients, 462 in total) showed that CD74 positivity was significantly associated with lymph node metastasis. CD74 overexpression was associated with a higher likelihood of lymph node metastasis (OR of 1.93, 95% CI 1.27–2.95; Z = 3.06, *p* = 0.002). No statistical heterogeneity was observed across the included studies (Chi^2^ = 0.91, df = 3, *p* = 0.82; I^2^ = 0%), reinforcing the consistency of the pooled estimate. The random-effects meta-analysis demonstrated the same association (OR of 1.94, 95% CI 1.27–2.96; Z = 3.08, *p* = 0.002), with consistent direction across all studies, ranged from OR = 1.62 (Zhang 2021-R2 [14]) to OR = 3.10 (Koide 2006 [11]), as shown in Figure 4. It is notable that no individual cohort reached statistical significance in isolation, so the association emerges only on pooling.

#### 3.2.4. Vascular Permeation

A fixed-effects meta-analysis of three studies (Koide 2006 [11], Nagata 2009 [12], Zhang 2014 [13]; including 105 CD74+ and 76 CD74- patients, 181 in total) evaluated the association between CD74 expression and vascular permeation. The pooled OR was 1.98 (95% CI 0.83–4.68; Z = 1.55, *p* = 0.12), suggesting a non-significant trend toward higher vascular permeation in CD74-positive tumors. No statistical heterogeneity was detected among the studies (Chi^2^ = 0.54, df = 2, *p* = 0.76; I^2^ = 0%). The random-effects meta-analysis obtained the same results (pooled OR was 1.99, 95% CI 0.84–4.68; Z = 1.57, *p* = 0.12), as shown in Figure 5. Although CD74-positive patients showed roughly twice the odds of vascular permeation, the limited number of studies and patients may have reduced the statistical power to detect a significant effect.

#### 3.2.5. Advanced pTNM Stage

A fixed-effects meta-analysis of three studies (Nagata 2009 [12], Zhang 2014 [13], Zhang 2021-R2 [14]; 153 CD74+ and 131 CD74- patients, 284 in total) evaluated the relationship between CD74 expression and advanced pTNM stage. The pooled OR was 1.62 (95% CI: 0.93–2.82; Z = 1.72, *p* = 0.09), indicating a non-significant trend toward higher pTNM stage among CD74-positive patients. No statistical heterogeneity was detected (Chi^2^ = 0.20, df = 2, *p* = 0.90; I^2^ = 0%). The random-effects meta-analysis obtained the same results (pooled OR was 1.62, 95% CI 0.93–2.82; Z = 1.72, *p* = 0.09), as shown in Figure 6. These results suggest a possible association between CD74 positivity and advanced disease stage that did not reach statistical significance, possibly due to the limited sample size. This analysis should be interpreted with particular caution because the contrast used to define advanced stage differs between cohorts: stages III+IV versus I+II in Nagata 2009 [12], stages III versus I+II in Zhang 2021-R2 [14], and stage II versus stage I in Zhang 2014 [13], which by design enrolled only stages I–II patients. The pooled estimate therefore describes a general association between CD74 positivity and more advanced stage rather than a single, uniformly defined contrast. No overlapping patients were included in the current meta-analysis between the two reports from Zhang 2014 [13] and Zhang 2021 [14].

#### 3.2.6. Association of CD74 with Overall Survival

A fixed-effects meta-analysis of three cohorts (Hong 2023 [6], Nagata 2009 [12], Zhang 2014 [13]; 293 patients) evaluated the association between CD74 expression and overall survival, using log[HR] and its standard error as inputs. The three estimates differ systematically in provenance, and the analysis was therefore structured as two subgroups. Nagata 2009 [12] and Zhang 2014 [13] assessed CD74 protein by immunohistochemistry, with thresholds of ≥70% and ≥25% stained cells respectively, and both contributed multivariable-adjusted hazard ratios reported in the original publications. Hong 2023 [6] assessed CD74 at transcript level in TCGA data accessed through GEPIA2 using the default median expression cut-off and reported no effect estimate; its hazard ratio was reconstructed from the published Kaplan–Meier curve by digitization (WebPlotDigitizer 4.7) and the method from Tierney et al., and is therefore univariable. Because the measurement modality and the derivation of the estimate differ together, these two features are completely confounded in this dataset and cannot be separated; the subgroups are labeled accordingly, and the modality, source and level of adjustment of each cohort are set out in Table 4.

In the immunohistochemistry subgroup, the pooled HR was 2.21 (95% CI 1.50–3.25; Z = 4.03, *p* < 0.0001), with no heterogeneity between the two cohorts (τ^2^ = 0.00; Chi^2^ = 0.71, df = 1, *p* = 0.40; I^2^ = 0%). The transcript-level estimate pointed in the same direction but did not reach statistical significance on its own (HR of 1.52, 95% CI 0.72–3.21; Z = 1.10, *p* = 0.27). Across all three cohorts the pooled HR was 2.04 (95% CI 1.45–2.88; Z = 4.09, *p* < 0.0001), indicating that CD74-negative patients had better overall survival than CD74-positive patients. The immunohistochemistry subgroup carried 78.9% of the weight and the transcript-level cohort by 21.1%. No statistical heterogeneity was detected (τ^2^ = 0.00; Chi^2^ = 1.47, df = 2, *p* = 0.48; I^2^ = 0%), and the test for subgroup differences was not significant (Chi^2^ = 0.76, df = 1, *p* = 0.38; I^2^ = 0%), as shown in Figure 7. Because the between-study variance was estimated as zero, the DerSimonian–Laird random-effects estimates were identical to the fixed-effects estimates reported above, both overall and within the immunohistochemistry subgroup.

**Figure 7:**
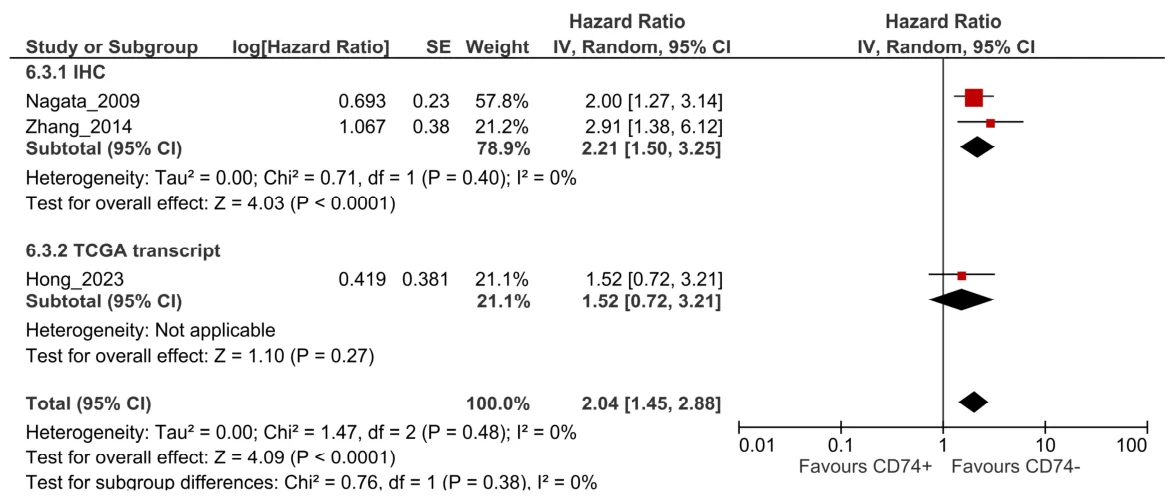
Forest plot of the association between CD74 expression and overall survival in patients with PDAC. Hong_2023 [6], Nagata_2009 [12], Zhang_2014 [13].

## 4. Discussion

To our knowledge, this is the first design-restricted meta-analysis to synthesize the reported associations of CD74 with clinicopathological and prognostic endpoints across human pancreatic neoplasms. Pooling five studies (six cohorts), we found that CD74 overexpression was significantly associated with perineural invasion, lymphatic invasion, lymph node metastasis, and shorter overall survival, while showing non-significant positive trends for vascular permeation and advanced pTNM stage. The direction of effect was consistent across all endpoints and no analysis showed statistical heterogeneity (I^2^ = 0% and τ^2^ = 0.00 throughout), so that fixed-effects and random-effects estimates were almost identical for every outcome. This consistency should not be over-read. With only three or four cohorts per endpoint, tests of heterogeneity have very limited power, and an I^2^ of 0% indicates only that the observed effect estimates are statistically compatible with one another; it says nothing about whether the underlying studies are clinically or methodologically comparable, which in this literature study they plainly are not. Collectively, these data position CD74 as a candidate marker of aggressive biological behavior and adverse prognosis in pancreatic cancer, while supporting an association rather than an established, independent prognostic role.

CD74, the MHC class II invariant chain, is a type II transmembrane glycoprotein that guides antigen presentation in immune cells [15]. In the endoplasmic reticulum it trimerises and binds newly synthesized MHC class II αβ heterodimers, occupying the peptide-binding groove with its CLIP segment and preventing premature peptide loading, then routes the complex through the endolysosomal pathway, where sequential proteolysis and HLA-DM-mediated CLIP exchange complete class II maturation. In its cell-surface form CD74 additionally functions as an accessory receptor that controls B-cell and dendritic-cell maturation, motility and survival through NF-κB-dependent signaling. Its best-characterized function in cancer is as the high-affinity receptor for macrophage migration inhibitory factor (MIF), usually in complex with CD44, transducing PI3K/AKT, ERK/MAPK and NF-κB signals that support proliferation, survival, motility and immune evasion. In PDAC, CD74 is expressed in tumor and stromal cells, and its signaling, acting as a cell-surface cytokine, contributes to the immunosuppressive, desmoplastic tumor microenvironment [16].

An important consequence of this dual biology is that CD74 is not a tumor-cell-restricted marker. In PDAC it is expressed by carcinoma cells but also by infiltrating antigen-presenting cells, macrophages and antigen-presenting cancer-associated fibroblasts [3]. This matters for the interpretation of our pooled estimates, because the meta-analysis necessarily treats CD74 positivity as a single biological variable when it is not. The four immunohistochemistry-based cohorts all expressed positivity as the percentage of stained carcinoma cells, using lymphocytes as an internal positive control, so they do target the tumor-cell compartment; none, however, reports a formal quantification of immune or stromal CD74, and the GEPIA2/TCGA estimate used by Hong 2023 [6] reflects bulk-tissue transcript abundance and therefore aggregates tumor, immune and stromal contributions. The same nominal score may thus reflect tumor-intrinsic MIF–CD74 signaling in one setting and a class II-rich immune infiltrate in another, and these two situations could carry opposite prognostic meaning, which is one plausible explanation for the context-dependent direction of the CD74 effect reported across tumor types. Cell-type-resolved quantification, for example by multiplex immunofluorescence or single-cell transcriptomics, will be needed before the pooled association can be attributed to any one cellular compartment.

Our pooled analysis showed that CD74 positivity was associated with a more than three-fold increase in the odds of perineural invasion (OR of 3.72, 95% CI 2.30–6.02), with all four contributing studies concordant in both direction and magnitude and no detectable heterogeneity (I^2^ = 0%). The absence of heterogeneity is noteworthy, given the variability that commonly affects immunohistochemistry-based biomarker meta-analyses, but it should not be over-interpreted. With four small cohorts, Cochran’s Q and I^2^ have very low power, and an I^2^ of 0% neither demonstrates biological homogeneity nor excludes clinically relevant heterogeneity arising from differences in antibody, staining protocol, scoring system, positivity threshold, patient population and, in this instance, the definition of perineural invasion itself. What can reasonably be said is that the four estimates were concordant in direction and in magnitude despite these methodological differences, which makes an artifact of any single scoring practice a less likely explanation for the association. This consistency is further supported by the mechanistic literature. CD74, the MHC class II invariant chain, was first implicated in pancreatic perineural invasion by Koide et al., who identified it through transcriptomic comparison of high- versus low-PNI cell lines in murine models and confirmed its upregulation in surgical specimens. Functionally, CD74 serves as the high-affinity receptor for macrophage migration inhibitory factor (MIF) [17], and MIF–CD74 engagement—frequently in concert with CD44 as a co-receptor [18]—activates downstream PI3K/AKT, ERK, and NF-κB signaling that drives a pro-migratory, pro-invasive phenotype [19]. More directly relevant to perineural spread, CD74 has been shown to potentiate tumor-cell migration and invasion while promoting secretion of glial cell line-derived neurotrophic factor (GDNF) through an AKT/EGR-1/GDNF axis, thereby enhancing neuroplasticity and establishing a reciprocal signaling loop between malignant cells and peripheral nerves [14].

CD74 positivity was also significantly associated with regional lymph node metastasis, though the effect was more moderate than that observed for perineural invasion, with a pooled odds ratio of 1.93 (95% CI 1.27–2.95, *p* = 0.002). It is worth noting that none of the contributing studies reached statistical significance in isolation—each confidence interval crossed unity—so the significant association emerges only on pooling. The experimental literature most strongly implicates CD74 in the neurotropic behavior of PDAC, whereas its contribution to nodal dissemination is likely mediated through more general pro-metastatic programs. As the receptor for macrophage MIF, CD74 activates PI3K/AKT, ERK, and NF-κB signaling that drives epithelial–mesenchymal transition, motility, and matrix invasion, and functional studies have shown that silencing CD74 in pancreatic cancer cells reduces proliferation, migration, and invasion while increasing apoptosis [6]. CD74 has also been reported to promote a pro-inflammatory, immunosuppressive tumor microenvironment—for example, by inducing secretion of S100A8 and S100A9—that may further facilitate the multistep process of lymphatic invasion and colonization. Our analysis also showed a significant association between CD74 positivity and lymphatic invasion (OR of 2.05, 95% CI 1.22–3.46, *p* = 0.007; I^2^ = 0%). Thus, with respect to lymphatic permeation, MIF, acting via CD44/CD74 and MAPK signaling, has been shown to upregulate vascular endothelial growth factor-C (VEGF-C), the principal lymphangiogenic factor whose expression closely correlates with lymphatic invasion and lymph node metastasis in several solid tumors [20,21]. Consistent with a pro-invasive function, silencing CD74 in pancreatic cancer cells reduces their migratory and invasive capacity. Collectively, the literature supports a mechanistically plausible link between CD74 and lymphatic invasion, although the estimate rests on four small retrospective cohorts that applied different definitions of both lymphatic invasion and CD74 positivity, so it should be regarded as suggestive and warrants confirmation in larger, prospectively scored cohorts.

These mechanisms provide a plausible basis for the observed association, positioning CD74 as a marker of broader metastatic competence in addition to its role in perineural spread. The pooled estimate indicated a significantly higher risk of death among CD74-positive patients compared with CD74-negative patients (pooled HR of 2.04, 95% CI 1.45–2.88, *p* < 0.0001), with all three cohorts pointing in the same direction and no detectable heterogeneity (I^2^ = 0%). The estimate was essentially unchanged when restricted to the two cohorts contributing multivariable-adjusted, immunohistochemistry-based hazard ratios (HR of 2.21, 95% CI 1.50–3.25), and the test for subgroup differences was not significant (*p* = 0.38), so the association does not appear to depend on the inclusion of the transcript-level dataset. This is an association, and it is not the same as establishing independent prognostic value. Two of the three contributing estimates are multivariable-adjusted, which is a strength, but they were adjusted for different variable sets; neither model included tumor grade or resection margin status; both were built by stepwise selection in small samples, and the third estimate is unadjusted, reconstructed from a published survival curve and derived from bulk transcriptomic data rather than tissue staining. Pooling estimates with heterogeneous adjustment sets does not produce an interpretable adjusted hazard ratio. Because CD74 positivity is itself associated with nodal metastasis and perineural invasion in these same cohorts, part of the observed survival difference may still reflect the more advanced disease of CD74-positive patients rather than an independent contribution of CD74. Confirming independent prognostic value would require multivariable, patient-level analyses using a common adjustment set, which the published literature does not currently provide. This prognostic signal is coherent with the preceding analyses: the same biology that links CD74 to perineural invasion, nodal metastasis, and lymphatic permeation would be expected to translate into worse survival, since these invasive and disseminative phenotypes are themselves principal determinants of outcome in pancreatic cancer. At the molecular level, MIF–CD74 signaling not only promotes migration and invasion but also sustains proliferative and anti-apoptotic programs—silencing CD74 in pancreatic cancer cells reduces proliferation and increases apoptosis—and contributes to an immunosuppressive tumor microenvironment, all of which plausibly compound to drive disease progression.

CD74 positivity showed a consistent positive trend toward both vascular invasion and more advanced TNM stage in PDAC, although neither association reached statistical significance. In both analyses, the confidence intervals crossed unity, and the contributing studies were small, so these results are best read as suggestive rather than confirmatory. These trends are mechanistically relevant. As the receptor for macrophage migration inhibitory factor (MIF), CD74—with its co-receptor CD44—activates ERK/MAPK, PI3K/AKT, and NF-κB signaling that drives epithelial–mesenchymal transition, motility, and matrix invasion, and silencing CD74 in pancreatic cancer cells reduces their migratory and invasive capacity. The MIF/CD74 axis is additionally pro-angiogenic: MIF enhances endothelial proliferation, correlates with tumor microvessel density, and can upregulate VEGF-family factors, offering a plausible route by which CD74 might specifically facilitate vascular permeation [22].

If CD74 is to be pursued as more than a descriptive marker, the therapeutic landscape has to be considered explicitly. Two broad strategies exist. The first targets CD74 directly: milatuzumab, a humanized anti-CD74 monoclonal antibody, has been evalu-ated in CD74-expressing hematological malignancies [23], and antibody–drug conju-gates such as STRO-001 exploit the rapid internalization and recycling of surface CD74 to deliver a cytotoxic payload [5]. The second targets the ligand: small-molecule MIF tautomerase inhibitors, such as ISO-1 and 4-IPP, and MIF-neutralizing antibodies disrupt MIF–CD74 engagement upstream of PI3K/AKT, ERK and NF-κB signaling [24]. Neither strategy has been tested prospectively in PDAC. The obstacles are substantial: surface CD74 represents only a small fraction of total cellular CD74; expression is shared with normal antigen-presenting cells, so on-target, off-tumor toxicity is a realistic concern; blocking a receptor with physiological roles in antigen presentation could in principle blunt antitumor immunity. Before clinical translation could be contemplated, the field would need cell-type-resolved quantification of CD74 in PDAC tissue, a standardized and analytically validated scoring assay, evidence that CD74 expression predicts benefit from CD74- or MIF-directed agents rather than merely marking a poor prognosis, and early-phase safety data in solid tumors. The present meta-analysis supplies none of that evidence; it establishes only that study-defined CD74 positivity marks a more aggressive clinicopathological phenotype.

Several limitations must temper these conclusions. First, and most importantly, the evidence base is small: only five studies met the inclusion criteria, each contributing modest patient numbers, and individual endpoints were informed by only two or three cohorts. With fewer than ten studies, small-study effects and publication bias could not be formally assessed (e.g., by funnel-plot asymmetry or Egger’s test), and the possibility that negative findings remain unpublished cannot be excluded. It bears emphasis that the inability to test for small-study effects is not evidence of their absence; with the literature this small, selective publication of positive biomarker associations remains a realistic possibility, and the pooled estimates may be biased away from the null. The small number of contributing cohorts also means that each pooled estimate is dominated by one or two datasets that apparently narrow confidence intervals and overstate the true precision of the evidence base, and that a single unpublished or future negative cohort could materially change the pooled result. Second, although heterogeneity was statistically absent (the LN-2 mouse monoclonal in the study by Koide 2006 [11] and the Dako mouse monoclonal in the study by Nagata 2009 [12]; Abcam preparations, including the rabbit clone ab108393, by Zhang 2014 [13] and Zhang 2021 [14]) in staining and scoring protocols and in the cut-offs used, positivity was defined (≥10%, ≥25% or ≥70% stained cells, and a median transcript-level cut-off in the GEPIA2-derived dataset). These categories are not necessarily biologically interchangeable, so a patient classified as CD74-positive in one cohort might be classified as negative in another. The ≥70% threshold used by Nagata 2009 [12] was, moreover, selected because it maximized prognostic separation, with other thresholds examined but not reported, which is likely to bias that estimate away from the null. This measurement heterogeneity is not captured by an I^2^ of 0%, and the absence of a standardized, validated threshold is in our view the single most important obstacle to the clinical use of CD74. The definitions of the outcomes also varied: Koide 2006 [11] compared invasion grade 2–3 with grade 0–1 rather than present with absent, and the contrast used for advanced pTNM stage differed across all three contributing cohorts. Third, all analyses were derived from unadjusted, study-level dichotomous data; we were unable to account for confounders such as tumor grade, resection margin status, or adjuvant therapy. For overall survival, two of the three contributing estimates were multivariable-adjusted, but they were adjusted for different variable sets and neither included tumor grade or resection margin status, while the third was univariable and was reconstructed from a published Kaplan–Meier curve; the pooled hazard ratio therefore cannot be interpreted as a properly adjusted effect, which limits causal inference. We addressed this by analyzing the survival data in subgroups defined by measurement modality and derivation of the estimate, but the two features are completely confounded in the available literature and the subgroup test has almost no power with a single cohort in one arm. Fourth, all included cohorts originate from a small number of East Asian centers in Japan, China and South Korea, and all are retrospective. Generalizability to European, North American and other populations is therefore uncertain, since risk-factor profiles, tumor biology, resection rates, adjuvant treatment pathways and follow-up intensity differ between these settings, and the prevalence of CD74 positivity itself ranged from 31% to 78% across the included cohorts. Patient totals also differed across endpoints owing to incomplete reporting within individual studies. Fifth, the cohorts differ in the treatment received before the stained specimen was obtained: every patient in the study by Nagata 2009 [12] received neoadjuvant chemoradiation and was resected only in the absence of interval progression, whereas the other cohorts were treatment-naive at resection, so both CD74 expression and the invasion endpoints were assessed after treatment in one of the two largest contributors to the perineural invasion analysis. Sixth, because Zhang 2014 [13] and Zhang 2021-R1 [14] recruited cases from the same institution over overlapping calendar periods and individual patient identifiers were not published, partially overlapping patients between these two datasets cannot be formally excluded, although the two were never pooled within the same analysis. Finally, while the review aimed to encompass diagnostic associations and the full range of pancreatic neoplasm subtypes, the available quantitative data were confined to the clinicopathological and prognostic endpoints reported above; diagnostic accuracy and subtype-specific effects could not be meta-analyzed.

These limitations define a clear agenda for future research. Larger, multicenter cohorts with standardized CD74 scoring and pre-specified positivity thresholds are needed to confirm the associations reported here and to narrow the confidence intervals for nodal status, lymphatic invasion, and the currently non-significant endpoints. Studies reporting multivariable-adjusted hazard ratios would strengthen the prognostic evidence, and analyses stratified by pancreatic neoplasm subtype would clarify whether the CD74 effect is uniform across histologies. Ultimately, prospective validation and, where appropriate, early-phase evaluation of CD74- or MIF-directed therapeutics will be required to translate this biomarker signal into clinical benefit.

## 5. Conclusions

This systematic review and meta-analysis, the first to specifically examine CD74 in pancreatic ductal adenocarcinoma, indicate that study-defined high- or positive-CD74 expression is associated with perineural invasion, lymphatic invasion, lymph node metastasis, and worse overall survival, with a consistent effect direction across all pooled endpoints and no statistically detectable heterogeneity, although the power to detect heterogeneity was very low and statistical consistency does not imply clinical comparability. Non-significant positive trends were also observed for vascular permeation and advanced pTNM stage, suggesting a broader role for CD74 in promoting aggressive tumor biology. These findings are mechanistically coherent with the established role of the MIF–CD74 signaling axis in driving cancer cell invasion, neuroplasticity, epithelial–mesenchymal transition, and immunosuppressive remodeling of the tumor microenvironment. However, the evidence base remains limited by a small number of retrospective, predominantly East Asian, single-center studies with heterogeneous IHC scoring thresholds and absence of multivariable-adjusted survival data. These results should be read as hypothesis-generating. They do not establish CD74 as an independent prognostic factor, nor do they demonstrate clinical utility for risk stratification or for therapeutic decision-making; the strongest and most consistent signal, the association with perineural invasion, is the one that most clearly merits further investigation. Larger, multicenter prospective studies using a standardized and analytically validated CD74 assay, applying uniform outcome definitions, reporting multivariable-adjusted hazard ratios from a common adjustment set and quantifying CD74 in defined cellular compartments are required before CD74 could be considered a clinically actionable biomarker, and dedicated preclinical and early-phase work will be needed to determine whether the MIF–CD74 axis is a tractable therapeutic target in PDAC.

## Figures and Tables

**Figure 1:**
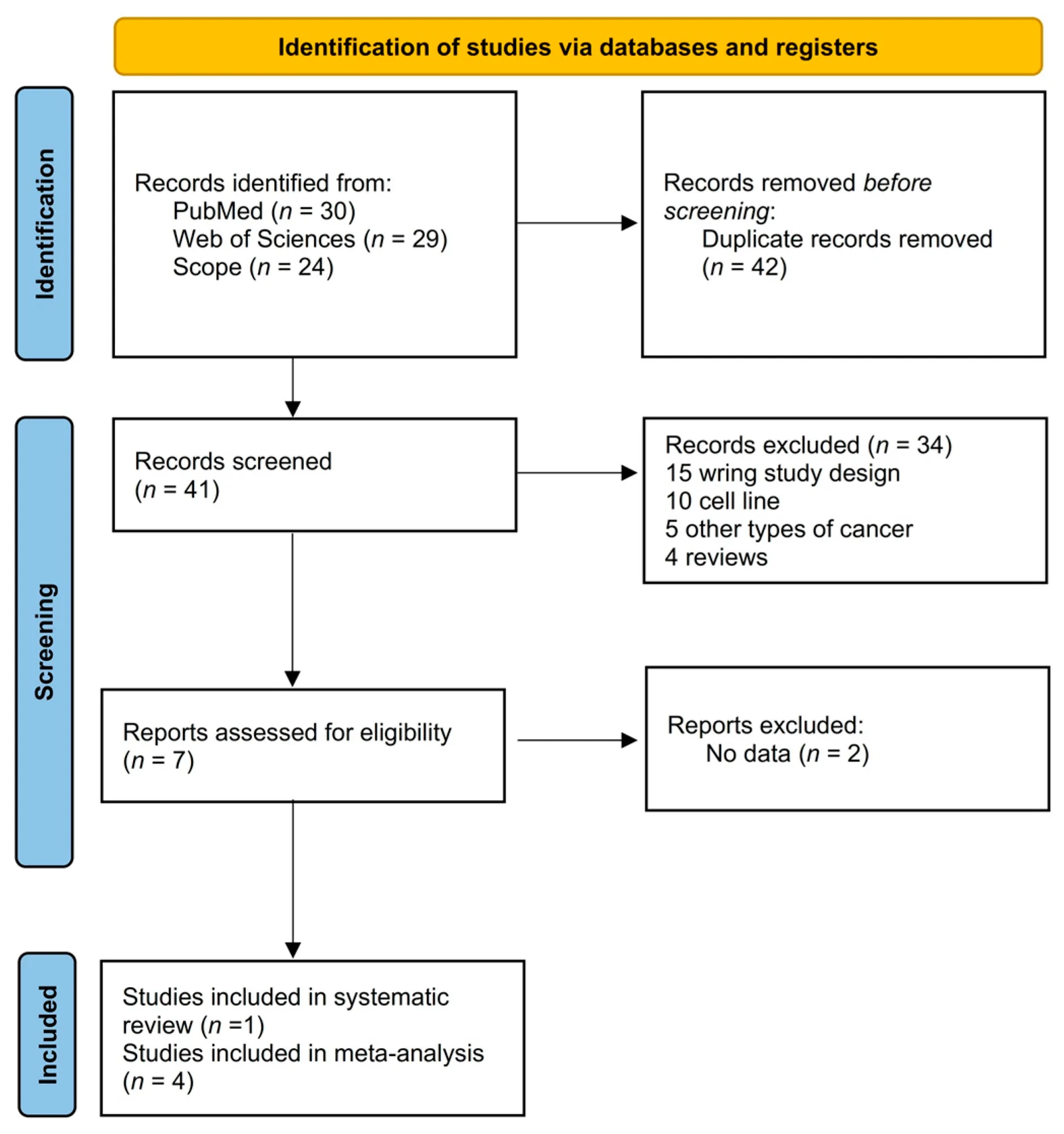
PRISMA flow diagram of the study selection process.

**Figure 2:**
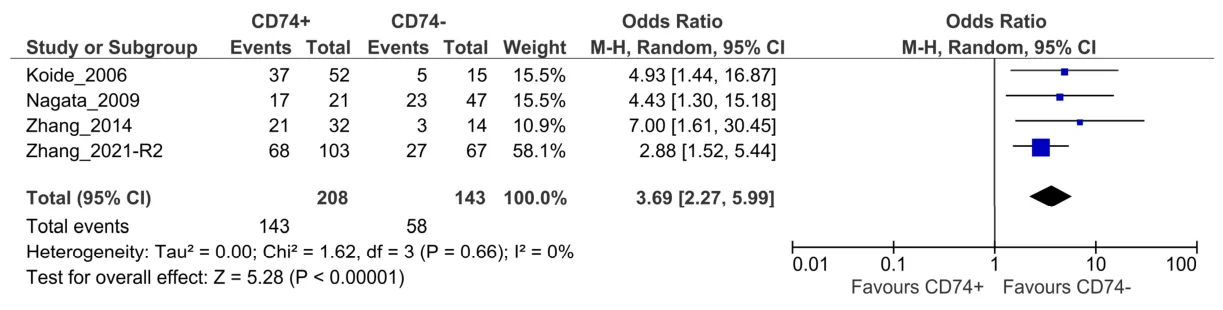
Forest plot of the association between CD74 expression and perineural invasion in patientswith PDAC. Koide_2006 [11], Nagata_2009 [12], Zhang_2014 [13], Zhang_2021_R2 [14].

**Figure 3:**
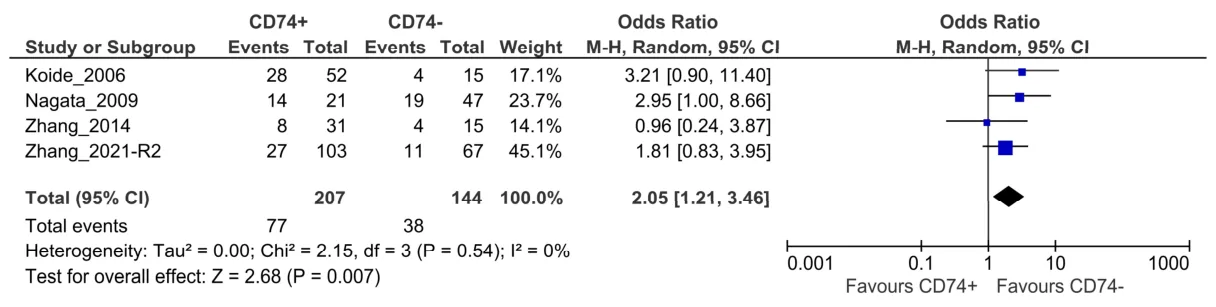
Forest plot of the association between CD74 expression and lymphatic invasion in patientswith PDAC. Koide_2006 [11], Nagata_2009 [12], Zhang_2014 [13], Zhang_2021_R2 [14].

**Figure 4:**
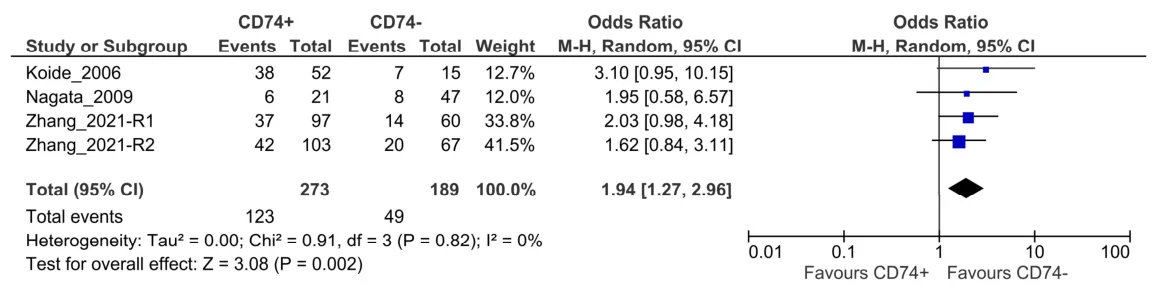
Forest plot of the association between CD74 expression and lymph node metastasis (pN) in patients with PDAC. Koide_2006 [11], Nagata_2009 [12], Zhang_2021_R1 [14], Zhang_2021_R2 [14].

**Figure 5:**
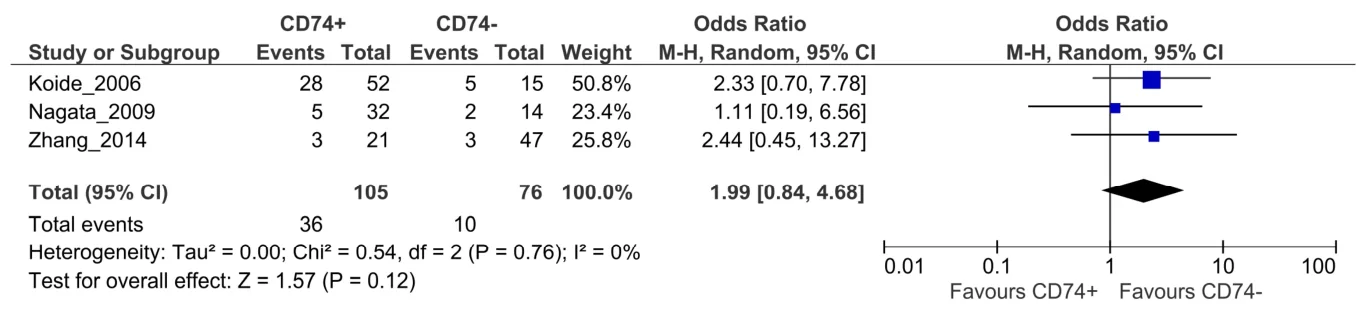
Forest plot of the association between CD74 expression and vascular permeation in patientswith PDAC. Koide_2006 [11], Nagata_2009 [12], Zhang_2014 [13].

**Figure 6:**
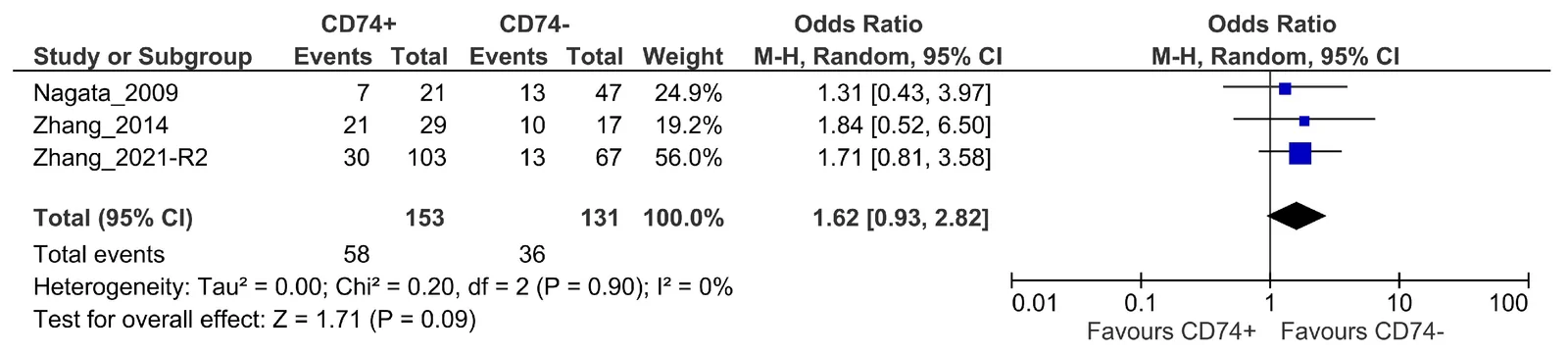
Forest plot of the association between CD74 expression and advanced pathological TNMstage (pTNM) in patients with PDAC. Nagata_2009 [12], Zhang_2014 [13], Zhang_2021_R2 [14].

**Table 1:** Statistical methods, effect measures and indices used in the meta-analysis.

Analytical Step	Method or Statistic	Specification
Software	Review Manager (RevMan) 5.4.1	Cochrane Collaboration, 2020, Copenhagen, Denmark
Effect measure, dichotomous outcomes	Odds ratio (OR) with 95% CI	Mantel–Haenszel method
Effect measure, survival	Hazard ratio (HR) with 95% CI	Generic inverse variance, using log[HR] and its SE
SE derivation	SE[log(HR)] = (ln(upper CI) − ln(lower CI))/3.92	Applied when HR and 95% CI were reported but SE was not
HR reconstruction	Tierney et al. method	From Kaplan–Meier curves digitized with WebPlotDigitizer 4.7
Test of heterogeneity	Cochran’s Q (Chi^2^ statistic)	*p* ≤ 0.10 is considered significant
Quantification of heterogeneity	I^2^ statistic and τ^2^	I^2^ < 25%, low; 25–50%, moderate; 50–75%, substantial; >75%, considerable
Primary pooling model	Fixed effects (Mantel–Haenszel; inverse variance for survival)	Applied because I^2^ = 0% and τ^2^ = 0.00 for every outcome
Secondary model	DerSimonian–Laird random effects	Computed for every outcome irrespective of I^2^
Test of overall effect	Z-test	Two-sided; *p* < 0.05 is considered statistically significant
Sensitivity analyses	Random-effects re-analysis; leave-one-out	Applied to every outcome
Subgroup analyses	By measurement modality and derivation of the estimate (survival)	Cut-off and geographic subgroups planned but not feasible (too few studies)
Publication bias	Funnel plot and Egger’s test	Not performed (<10 studies per outcome)
Risk of bias	QUIPS, six domains	Rated low, moderate or high per domain (Supplementary File S3)
Certainty of evidence	GRADE adapted for prognostic factors	Rated per outcome (Supplementary File S4)
Presentation	Forest plots	Individual and pooled estimates with 95% CIs

**Table 2:** Main characteristics of the five studies (six cohorts) included in the meta-analysis of CD74 expression in pancreatic ductal adenocarcinoma.

Study	Country	Period	Patients’ CD74 (Positive Rate)	Design	CD74 Detection Method	Cut-off CD74+
Hong 2023 [6]	South Korea	TCGA PAAD cohort	179 TCGA PAAD tumors; dichotomised at the GEPIA2 median (no case-level positivity rate reported)	Experimental (in vitro/in vivo) with survival analysis based on publicly available TCGA data (GEPIA2 database)	mRNA expression via GEPIA2 database (for survival analysis); IHC, qRT-PCR, and Western blot (for tissue validation)	Median expression level (GEPIA2 default)
Koide 2006 [11]	Japan	1991–2004	52/67 (77.6%)	Retrospective clinicopathological study with experimental in vivo perineural invasion models	IHC using anti-CD74 mouse monoclonal antibody (LN-2, Dako), dilution of 1:100	≥10% cells = pos
Nagata 2009 [12]	Japan	11.2002–10.2007	21/68 (30.9%)	Retrospective cohort, prognostic IHC (multimodal therapy)	IHC using anti-CD74 mouse monoclonal antibody (Dako A/S), dilution of 1:100, streptavidin–biotin labeling method	≥70% cells = Level II (high)
Zhang 2014 [13]	China	01.2008–12.2009	32/46 (69.6%) Stage restriction: exclusively included stages I and II patients (per AJCC 7th edition)	Retrospective cohort, prognostic IHC (resectable stages I-II)	IHC using mouse monoclonal anti-CD74 antibody (Abcam), concentration of 1% (*v*/*v*)	≥25% cells = pos
Zhang 2021-R1 [14]	China	01.2002–06.2013	97/157 (61.8%)	Retrospective cohort study with experimental validation	IHC on tissue microarray using rabbit anti-CD74 antibody (Abcam, ab108393), dilution of 1:200	≥25% positive cells = CD74-positive
Zhang 2021-R2 [14]		07.2013–12.2016	103/170 (60.6%)			

GEPIA, Gene Expression Profiling Interactive Analysis; IHC, immunohistochemistry; mRNA, messenger RNA; qRT-PCR, quantitative reverse-transcription polymerase chain reaction; R1, Renji cohort 1 from Zhang et al. 2021 [14]; R2, Renji cohort 2 from Zhang et al. 2021 [14]; TCGA, The Cancer Genome Atlas.

**Table 3:** Pooled effects for all outcomes.

Study	Perineural Invasion	Lymphatic Invasion	pN Status	Vascular Permeation	pTNM Stage	OS
Hong 2023 [6]						Y
Koide 2006 [11]	Y	Y	Y	Y		
Nagata 2009 [12]	Y	Y	Y	Y	Y	Y
Zhang 2014 [13]	Y	Y		Y	Y	Y
Zhang 2021-R1 [14]			Y			
Zhang 2021-R2 [14]	Y	Y	Y		Y	
Fixed Effects Pooled	3.72 [2.30–6.02]	2.05 [1.22–3.46]	1.93 [1.27–2.95]	1.98 [0.83–4.68]	1.62 [0.93–2.82]	1.85 [1.35, 2.53]
Random Effects Pooled	3.69 [2.27, 5.99]	2.05 [1.21, 3.46]	1.94 [1.27, 2.96]	1.99 [0.84, 4.68]	1.62 [0.93, 2.82]	2.04 [1.45, 2.88]
Heterogeneity	*p* = 0.66; I^2^ = 0%	*p* = 0.54; I^2^ = 0%	*p* = 0.82; I^2^ = 0%	*p* = 0.76; I^2^ = 0%	*p* = 0.90; I^2^ = 0%	*p* = 0.48; I^2^ = 0%

**Table 4:** Provenance, adjustment and magnitude of the hazard ratios contributing to the overall survival meta-analysis.

Cohort	CD74 Measurement and Threshold	Source of the Hazard Ratio	Adjustment	HR (95% CI)	Patients
Hong 2023 [6]	CD74 mRNA; TCGA data accessed through GEPIA2; default median expression cut-off	External gene-expression database; log[HR] of 0.419, SE of 0.168 derived Kaplan–Meier survival curves	Unadjusted	1.52 (1.09–2.11)	179
Nagata 2009 [12]	CD74 protein; immunohistochemistry; ≥70% stained cells (level II)	Multivariable Cox model reported in the original article	Adjusted (stepwise) for vascular permeation, nodal status, pTNM stage and post-therapeutic CA19-9	2.00 (1.27–3.14)	68
Zhang 2014 [13]	CD74 protein; immunohistochemistry; ≥25% stained cells	Multivariable Cox model reported in the original article	Adjusted (stepwise) for lymphatic permeation, with age, perineural invasion and pTNM stage eligible for entry	2.91 (1.38–6.12)	46

## Data Availability

No new data were created or analyzed in this study. Data sharing is not applicable to this article.

## Supplementary Materials

The following supporting information can be downloaded at https://www.mdpi.com/article/10.3390/ijms27198488/s1.
