# Supplementary material for: Prognostic and Clinicopathological Significance of CD74 Expression in Pancreatic Ductal Adenocarcinoma: A Systematic Review and Meta-Analysis
Source: Int J Mol Sci. 2026 Sep 23;27(19):8488. doi: 10.3390/ijms27198488 (PMC13665301; doi:10.3390/ijms27198488)
Supplement: Supplementary file 1 [file ijms-27-08488-s001.zip › Suppl File S2.pdf]

SupplFile S2 – Search strategy for identifying studies reporting CD74 expression in pancreatic ductal adenocarcinoma (PDAC) and its association with prognosis.

Databases: PubMed/MEDLINE, Web of Science Core Collection, Scopus
Date range: Inception to May 20th, 2026
Language: No restriction at search stage (applied at screening)

PubMed

#1 "CD74 protein, human" [Supplementary Concept] OR "Antigens, Differentiation, B-Lymphocyte"[MeSH]
OR "Histocompatibility Antigens Class II"[MeSH]
#2 CD74[tiab] OR "CD-74"[tiab] OR "invariant chain"[tiab] OR "Ii chain"[tiab] OR "Ii antigen"[tiab]
OR "HLA-DR antigens-associated invariant chain"[tiab] OR "gamma chain"[tiab] OR DHLAG[tiab]
OR HLADG[tiab] OR "MIF receptor"[tiab] OR "p33 invariant chain"[tiab]
#3 #1 OR #2
#4 "Pancreatic Neoplasms"[MeSH] OR "Carcinoma, Pancreatic Ductal"[MeSH]
#5 pancrea*[tiab] AND (cancer*[tiab] OR neoplas*[tiab] OR carcinoma*[tiab] OR adenocarcinoma*[tiab]
OR tumo?r*[tiab] OR malignan*[tiab])
#6 PDAC[tiab] OR "pancreatic ductal adenocarcinoma"[tiab] OR "ductal adenocarcinoma of the pancreas"[tiab]
#7 #4 OR #5 OR #6
#8 #3 AND #7
#9 #8 NOT ("editorial"[pt] OR "comment"[pt] OR "letter"[pt] OR "case reports"[pt])
#10 #9 NOT ("animals"[MeSH] NOT "humans"[MeSH])

Web of Science Core Collection

#1 TS=(CD74 OR "CD-74" OR "invariant chain" OR "Ii chain" OR "Ii antigen" OR "MIF receptor" OR "HLA class II histocompatibility antigen gamma chain" OR DHLAG OR HLADG OR "p33 invariant chain") #2 TS=(pancrea* NEAR/3 (cancer* OR neoplas* OR carcinoma* OR adenocarcinoma* OR tumor* OR tumour* OR malignan*)) OR TS=(PDAC OR "pancreatic ductal adenocarcinoma") #3 #1 AND #2 #4 #3 NOT DT=(Editorial Material OR Letter OR Meeting Abstract OR Note)

Scopus

#1 TITLE-ABS-KEY(CD74 OR "CD-74" OR "invariant chain" OR "Ii chain" OR "Ii antigen" OR "MIF receptor" OR "HLA class II histocompatibility antigen gamma chain" OR DHLAG OR HLADG OR "p33 invariant chain") #2 TITLE-ABS-KEY(pancrea* W/3 (cancer* OR neoplas* OR carcinoma* OR adenocarcinoma* OR tumor* OR tumour* OR malignan*)) OR TITLE-ABS-KEY(PDAC OR "pancreatic ductal adenocarcinoma") #3 #1 AND #2 #4 #3 AND NOT DOCTYPE(re OR ed OR le OR no OR ch OR cp) #5 #4 AND (LIMIT-TO(EXACTKEYWORD, "Human") OR EXCLUDE(EXACTKEYWORD, "Animals"))
