# Supplementary material for: Prognostic and Clinicopathological Significance of CD74 Expression in Pancreatic Ductal Adenocarcinoma: A Systematic Review and Meta-Analysis
Source: Int J Mol Sci. 2026 Sep 23;27(19):8488. doi: 10.3390/ijms27198488 (PMC13665301; doi:10.3390/ijms27198488)
Supplement: Supplementary file 1 [file ijms-27-08488-s001.zip › Suppl File S3_QUIPS.pdf]

Supplementary Table S3. Study-by-study risk-of-bias assessment of the six included cohorts using the Quality in Prognosis Studies (QUIPS) tool.

Prognostic and Clinicopathological Significance of CD74 Expression in Pancreatic Ductal Adenocarcinoma: A Systematic Review and Meta-Analysis (SupplFile S3).

Each cohort was assessed independently by two reviewers (I.A.V. and A.T.-S.) across the six QUIPS domains, with disagreements resolved by consensus or by arbitration by a third reviewer (B.S.U.). Each domain was rated as low, moderate or high risk of bias, and a domain was rated not assessable where the cohort contributed no data of the type the domain addresses. Overall judgements are driven by the domains most likely to bias the pooled estimates in a prognostic-factor review, namely prognostic factor measurement, study confounding, and statistical analysis and reporting.

A. Traffic-light summary

Cohort	1. Participation	2. Attrition	3. PF measurement	4. Outcome measurement	5. Confounding	6. Analysis & reporting	Overall
**Hong 2023 [6]**	**− High**	**− High**	**− High**	**+ Low**	**− High**	**− High**	**− High**
**Koide 2006 [11]**	**? Moderate**	**n/a Not assessable**	**? Moderate**	**+ Low**	**− High**	**? Moderate**	**? Moderate**
**Nagata 2009 [12]**	**? Moderate**	**? Moderate**	**− High**	**+ Low**	**? Moderate**	**− High**	**− High**
**Zhang 2014 [13]**	**? Moderate**	**+ Low**	**? Moderate**	**+ Low**	**? Moderate**	**− High**	**? Moderate**
**Zhang 2021-R1 [14]**	**? Moderate**	**+ Low**	**? Moderate**	**+ Low**	**? Moderate**	**? Moderate**	**? Moderate**
**Zhang 2021-R2 [14]**	**? Moderate**	**+ Low**	**? Moderate**	**+ Low**	**? Moderate**	**? Moderate**	**? Moderate**

Legend: + Low risk of bias ? Moderate risk of bias − High risk of bias n/a Not assessable (the cohort contributed no data of this type).

B. Distribution of judgements by domain

QUIPS domain	Low	Moderate	High	Not assessable
1. Study participation	0 of 6	5 of 6	1 of 6	0 of 6
2. Study attrition	3 of 6	1 of 6	1 of 6	1 of 6
3. Prognostic factor measurement	0 of 6	4 of 6	2 of 6	0 of 6
4. Outcome measurement	6 of 6	0 of 6	0 of 6	0 of 6
5. Study confounding	0 of 6	4 of 6	2 of 6	0 of 6
6. Statistical analysis and reporting	0 of 6	3 of 6	3 of 6	0 of 6

Outcome measurement is the only domain rated at low risk throughout, because every endpoint was either overall survival or a feature determined by routine histopathological examination using stated criteria. Prognostic factor measurement and study confounding drove the overall judgements: no cohort used a validated or externally derived positivity threshold except Zhang 2021, which adopted the threshold of Zhang 2014, and none of the estimates extracted for the clinicopathological analyses is adjusted. Blinded scoring was explicitly reported by Nagata 2009 and Zhang 2021 only.

C. Domain-level judgements with supporting evidence

Cohort	QUIPS domain	Judgement	Evidence from the primary article
**Hong 2023 [6]**	1. Study participation	**High**	The survival estimate comes from TCGA PAAD data accessed through GEPIA2, a public resource not assembled to answer a prognostic question about CD74. No eligibility criteria, recruitment period, stage distribution, treatment details or baseline characteristics are reported for the patients contributing to the survival curves. The separate institutional series described in the Methods (30 patients resected at Gangnam Severance Hospital, 2018-2019) was used only for qRT-PCR, Western blot and representative immunostaining, and contributes nothing to the survival analysis.
	2. Study attrition	**High**	Follow-up duration, censoring pattern, losses to follow-up and the number of deaths are not reportable from the GEPIA2 output, and no comparison between patients with and without complete follow-up is possible.
	3. Prognostic factor measurement	**High**	CD74 was quantified as bulk-tissue mRNA and dichotomised by the GEPIA2 default median split, a data-derived threshold rather than a pre-specified, validated cut-off. Bulk transcript abundance aggregates carcinoma, immune and stromal CD74, and the measure is indirect for a review framed around tissue CD74 protein. The immunohistochemistry shown in the paper (Figure 1C) is illustrative only: no scoring system, threshold or case-level positivity rate is reported.
	4. Outcome measurement	**Low**	Overall survival is an objective endpoint recorded uniformly in the TCGA clinical files and applied identically to the high- and low-expression groups.
	5. Study confounding	**High**	No adjustment for stage, grade, nodal status, resection margin, adjuvant treatment or performance status. No covariates are reported at all for the survival cohort.
	6. Statistical analysis and reporting	**High**	The Statistical analysis section describes only one-way and two-way ANOVA in GraphPad Prism; the survival method is not described, no hazard ratio or confidence interval is reported for CD74, and only a log-rank p value ($p = 0.013$ for overall survival) appears in the figure. Any effect estimate for synthesis must therefore be reconstructed from the published curve.
	**Overall judgement**	**High**	*Determined by the highest-risk domains above.*
**Koide 2006 [11]**	1. Study participation	**Moderate**	Sixty-seven invasive ductal pancreatic adenocarcinomas resected at a single centre over a 13-year period (1991-2004), with ethics committee approval. Consecutive enrolment is not stated, only age and sex are reported as baseline characteristics, and restriction to resected specimens narrows the disease spectrum represented.
	2. Study attrition	**Not assessable**	The cohort contributed only cross-sectional clinicopathological comparisons; the paper reports no survival analysis and no follow-up data, so this domain cannot be rated within the QUIPS framework.
	3. Prognostic factor measurement	**Moderate**	Strengths: a widely used commercial antibody (LN-2, DAKO, 1:100), lymphocytes as an explicit internal positive control, scoring by three independent observers, and a stated threshold (>=10% positive cancer cells). Weaknesses: blinding of the observers to clinicopathological data is not stated, no inter-observer agreement statistic is reported, and the >=10% threshold is study-specific, is the lowest of any included cohort, and yields a very high positivity rate (52/67, 78%), leaving only 15 negative cases.
	4. Outcome measurement	**Low**	Histological diagnoses followed WHO criteria and the Japan Pancreas Society classification, and perineural invasion is defined unusually precisely, with epineural invasion and nerve involvement explicitly excluded. Note that invasion endpoints are graded 0-3 and dichotomised as 0-1 versus 2-3 rather than as absent versus present, so this cohort contributes a different outcome definition from the others.
	5. Study confounding	**High**	Associations are reported as simple cross-tabulations with no adjustment for tumour size, grade, stage, margin status or adjuvant treatment.
	6. Statistical analysis and reporting	**Moderate**	Chi-square testing across ten clinicopathological variables without correction for multiplicity and without a pre-specified analysis plan; no effect estimates or confidence intervals are reported, and the 78% positivity rate produces small and unstable cell counts in the CD74-negative group.
	**Overall judgement**	**Moderate**	*Determined by the highest-risk domains above.*

Cohort	QUIPS domain	Judgement	Evidence from the primary article
**Nagata 2009 [12]**	1. Study participation	**Moderate**	Sixty-eight patients resected at a single centre (November 2002 to October 2007) with IRB approval and full baseline reporting. The population is nevertheless treatment-defined: every patient received neoadjuvant chemoradiation (50 Gy plus gemcitabine) and was restaged, with only those free of progression proceeding to resection, followed by postoperative liver perfusion chemotherapy. Selection on absence of interval progression is a recognised source of bias and was explicitly criticised by Zhang et al. 2014.
	2. Study attrition	**Moderate**	Follow-up is described (survivors followed 4.6 to 61.5 months, median 20.1), but only 43 of 68 patients were followed for three years or until death while the remaining 25 were followed for less than three years, and 3- and 5-year rates are nevertheless reported. Ten patients died of causes other than the tumour and no comparison of completers with non-completers is given.
	3. Prognostic factor measurement	**High**	Strengths: a defined antibody (Dako A/S, 1:100), streptavidin-biotin detection, lymphocytes as an internal positive control, and scoring performed without knowledge of the clinicopathological factors (explicitly stated). Decisive weakness: the >=70% threshold defining level II was chosen because it maximised prognostic separation. The paper states that the prognostic significance was greatest at this cut-off and that thresholds of 30%, 50% and 90% were also examined but are not shown. An outcome-optimised, selectively reported threshold biases the effect estimate away from the null.
	4. Outcome measurement	**Low**	Overall survival, together with lymphatic permeation, perineural invasion, vascular permeation, nodal status and pTNM stage determined by two pathologists on standard sections, ascertained identically in both expression groups.
	5. Study confounding	**Moderate**	A stepwise multivariable Cox model was fitted and CD74 remained significant (relative risk 2.00, 95% CI 1.3-3.2, p = 0.003) alongside vascular permeation. Adjustment is partial: only variables significant on univariate testing were eligible for entry, and grade, resection margin and histological response to chemoradiation are not included.
	6. Statistical analysis and reporting	**High**	The reported cut-off was selected on the basis of the outcome with alternatives left unreported, and stepwise variable selection was applied to a small sample with 29 deaths, which risks overfitting. Effect estimates are otherwise reported clearly with confidence intervals.
	**Overall judgement**	**High**	*Determined by the highest-risk domains above.*
**Zhang 2014 [13]**	1. Study participation	**Moderate**	Forty-six patients receiving R0 resection at a single centre over two years (1 January 2008 to 31 December 2009), with ethics approval, full baseline reporting and no neoadjuvant treatment. The cohort is deliberately restricted to AJCC 7th-edition stage I-II resectable disease, so it represents resectable PDAC well but the wider PDAC population poorly, and the sample is the smallest of the six.
	2. Study attrition	**Low**	Follow-up is essentially complete: one perioperative death is reported explicitly, the remaining 45 patients were followed to death or to 31 December 2012, and the outcome of every patient is accounted for (35 cancer deaths, 3 other-cause deaths, 7 alive).
	3. Prognostic factor measurement	**Moderate**	A stated threshold (>=25% positive cancer cells), lymphocytes as an internal positive control and normal duct as a negative control. Weaknesses: the antibody clone is not specified and the concentration is reported as 1% (v/v) rather than as an interpretable dilution, blinded scoring is not stated, and the threshold is study-specific and not externally validated.
	4. Outcome measurement	**Low**	Overall survival with near-complete follow-up, plus perineural invasion, lymphatic permeation, vascular permeation and pTNM stage assessed by two senior pathologists according to WHO criteria, with perineural invasion explicitly defined.
	5. Study confounding	**Moderate**	A multivariable Cox model was fitted and identified CD74 expression and lymphatic permeation as independent prognostic factors (CD74: relative risk 2.907, 95% CI 1.9-11.3). Stage is partly controlled by the I-II restriction, but grade, resection margin and adjuvant compliance are not adjusted for, and the model is fitted to 38 deaths among 46 patients.
	6. Statistical analysis and reporting	**High**	Table 1 contains internal inconsistencies: in the lymphatic permeation row the CD74-negative counts sum to 15 and in the pTNM stage row to 17, although the CD74-negative group contains 14 patients. These are precisely two of the endpoints extracted for the present meta-analysis. P values are also reported as 0.000, and the multivariable confidence intervals are difficult to reconcile with the point estimates (relative risk 2.907 with 95% CI 1.9-11.3; relative risk 4.676 with 95% CI 1.4-6.2).
	**Overall judgement**	**Moderate**	*Determined by the highest-risk domains above.*

Cohort	QUIPS domain	Judgement	Evidence from the primary article
**Zhang 2021-R1 [14]**	1. Study participation	**Moderate**	A consecutive institutional cohort drawn from 205 radical resections performed between January 2002 and June 2013, reduced to 157 patients after excluding perioperative deaths, stage IV disease and patients without complete follow-up beyond two years. Recruitment window and exclusions are clearly stated, but the study is single-centre and selection on availability of two years of follow-up removes patients lost early.
	2. Study attrition	**Low**	By design only patients with complete follow-up over more than two years were enrolled, so loss to follow-up within the analysed cohort is minimal; the corresponding concern is carried by the participation domain rather than by attrition.
	3. Prognostic factor measurement	**Moderate**	A defined rabbit antibody (Abcam ab108393, 1:200) applied to tissue microarrays, a >=25% threshold taken from a previously published report rather than derived from these data, and scoring performed in a blinded manner by two experienced pathologists. Residual concerns: tissue microarray cores may under-sample heterogeneous staining, and no inter-observer agreement statistic is reported.
	4. Outcome measurement	**Low**	Lymph node metastasis, the endpoint this cohort contributes here, was taken from routine pathology reports classified according to the AJCC 8th edition and applied identically in both groups.
	5. Study confounding	**Moderate**	Univariate and multivariable Cox models including clinical stage, perineural invasion, tumour differentiation and tumour size are reported for survival. The clinicopathological cross-tabulations extracted for the present review are unadjusted.
	6. Statistical analysis and reporting	**Moderate**	Methods are appropriate to the design and both univariate and multivariable estimates are reported with confidence intervals. However, the clinicopathological correlations used here appear only in supplementary tables that could not be checked, and Figure 1C is headed 'Renji cohort 1' while the legend attributes panels B and C to cohort 2, leaving the source cohort of some estimates ambiguous.
	**Overall judgement**	**Moderate**	*Determined by the highest-risk domains above.*
