# Supplementary material for: Prognostic and Clinicopathological Significance of CD74 Expression in Pancreatic Ductal Adenocarcinoma: A Systematic Review and Meta-Analysis
Source: Int J Mol Sci. 2026 Sep 23;27(19):8488. doi: 10.3390/ijms27198488 (PMC13665301; doi:10.3390/ijms27198488)
Supplement: Supplementary file 1 [file ijms-27-08488-s001.zip › Suppl File S4_GRADE.pdf]

Supplementary Table S4. Outcome-level certainty of the body of evidence, assessed with the GRADE approach adapted for prognostic factor research.

Prognostic and Clinicopathological Significance of CD74 Expression in Pancreatic Ductal Adenocarcinoma: A Systematic Review and Meta-Analysis (Suppl File S4).

A. Method

Certainty was rated at outcome level following the adaptation of GRADE to prognostic factor research proposed by Huguet et al. (Syst Rev 2013;2:71) and the subsequent GRADE guidance of Foroutan et al. (J Clin Epidemiol 2020;121:62–70). Under this framework, a body of evidence assembled from adequately conducted prognostic factor studies begins at high certainty rather than at the low starting point used for observational studies of interventions, because a well-designed prognostic study is the appropriate design for a prognostic question. Certainty may then be downgraded by one or two levels in each of five domains — phase of investigation and study limitations, inconsistency, indirectness, imprecision, and publication bias — and upgraded for a large effect, a dose–response gradient, or for plausible residual confounding that would act against the observed association. No outcome was upgraded. Perineural invasion was the only candidate, its odds ratio of 3.72 meeting the usual threshold for a large effect, but GRADE advises against upgrading while residual concerns about bias remain, and that analysis is downgraded for indirectness and rests on cohorts rated at moderate to high risk of bias in the QUIPS assessment.

Two reviewers (I.A.V. and A.T.-S.) rated each domain independently, with disagreements resolved by consensus or by arbitration by a third reviewer (B.S.U.). Domain judgements draw directly on the study-by-study QUIPS assessment (Supplementary Table S3) and on the pooled estimates reported in Section 3.2. Ratings are expressed as high, moderate, low or very low; the lowest possible rating is very low, so further downgrading below that level has no effect.

B. Summary of findings and certainty ratings

Outcome	Cohorts	Patients (events)	Pooled effect (95% CI)	Study limitations	Inconsistency	Indirectness	Imprecision	Publication bias	Certainty
**Perineural invasion**	4	351 (201)	OR 3.72 (2.30–6.02)	Serious (−1)	Not serious	Serious (−1)	Not serious	Not serious	⊕⊕○○ **Low**
**Lymphatic invasion**	4	351 (115)	OR 2.05 (1.22–3.46)	Serious (−1)	Not serious	Serious (−1)	Serious (−1)	Not serious	⊕○○○ **Very low**
**Lymph node metastasis**	4	462 (172)	OR 1.93 (1.27–2.95)	Serious (−1)	Not serious	Serious (−1)	Serious (−1)	Not serious	⊕○○○ **Very low**
**Vascular permeation**	3	181 (46)	OR 1.98 (0.83–4.68)	Serious (−1)	Not serious	Serious (−1)	Very serious (−2)	Not serious	⊕○○○ **Very low**
**Advanced pTNM stage**	3	284 (94)	OR 1.62 (0.93–2.82)	Serious (−1)	Not serious	Very serious (−2)	Serious (−1)	Not serious	⊕○○○ **Very low**
**Overall survival**	3	293 (n.r.)	HR 2.04 (1.45–2.88)	Serious (−1)	Not serious	Serious (−1)	Not serious	Not serious	⊕⊕○○ **Low**

Certainty: ⊕⊕⊕⊕ High ⊕⊕⊕○ Moderate ⊕⊕○○ Low ⊕○○○ Very low. Events are the number of patients with the clinicopathological feature, summed across both CD74 groups; n.r. = deaths not reported uniformly across the three survival cohorts. Effect estimates are the fixed-effect estimates reported in Section 3.2; the corresponding random-effects estimates were almost identical ($\tau^2 = 0.00$ for every outcome).

Interpretation: low certainty means that the true association may differ substantially from the estimate, and very low certainty means that the estimate is very uncertain. No outcome reached moderate or high certainty, which is consistent with the conclusion in the manuscript that these findings are hypothesis-generating and that CD74 is not yet an established prognostic biomarker in PDAC.

C. Reasons for the domain judgements

Outcome	Domain	Judgement	Reason
**Perineural invasion**	Study limitations	**Serious (−1)**	Serious (−1). Three of the four contributing cohorts were rated at moderate overall risk of bias in the QUIPS assessment and one, Nagata 2009, at high risk; none adjusted the association; blinded scoring is reported by only two of the four; and all four applied a study-specific, non-validated positivity threshold. The downgrade is limited to one level because the high-risk cohort carries only 15.5% of the weight and its estimate (OR 4.43) is not an outlier.
	Inconsistency	**Not serious**	Not serious. All four estimates lie between OR 2.88 and OR 7.00, all four confidence intervals exclude unity, and there is no statistical heterogeneity (τ^2 = 0.00; Chi² = 1.62, df = 3, p = 0.66; I^2 = 0%).
	Indirectness	**Serious (−1)**	Serious (−1). Three sources of indirectness act together: all cohorts are East Asian and all are retrospective surgical series, so the evidence is indirect for the wider PDAC population; CD74 positivity is defined by four different, non-validated thresholds; and Koide 2006 contributed a comparison of perineural invasion grade 2–3 versus 0–1 rather than present versus absent, so the pooled estimate combines two related but non-identical contrasts.
	Imprecision	**Not serious**	Not serious. The analysis includes 351 patients and 201 events, and the 95% confidence interval (2.30–6.02) excludes unity with both bounds indicating an association of clinical importance.
	Publication bias	**Not serious**	Not serious, but suspected. Formal testing was not possible with fewer than ten studies. Against downgrading, perineural invasion was reported by all four cohorts as one item within a pre-specified panel of clinicopathological variables that also included null associations, so selective reporting within studies is unlikely. Selective publication of whole studies cannot be excluded and is acknowledged in the Limitations.
	**Certainty**	**⊕⊕○○ Low**	*Starting at high certainty for prognostic factor evidence, downgraded as set out above. No upgrade was applied, because residual concerns about bias and indirectness remain in every analysis.*
**Lymphatic invasion**	Study limitations	**Serious (−1)**	Serious (−1). The same risk-of-bias profile as for perineural invasion applies, but with the additional problem that the counts extracted from Zhang 2014 for this endpoint (31 CD74-positive and 15 CD74-negative patients) do not match that cohort's group sizes (32 and 14), an internal inconsistency in Table 1 of the primary article that directly affects the data entering this analysis.
	Inconsistency	**Not serious**	Not serious. No statistical heterogeneity (τ^2 = 0.00; Chi² = 2.15, df = 3, p = 0.54; I^2 = 0%) and a consistent direction in three of the four cohorts.
	Indirectness	**Serious (−1)**	Serious (−1). As for perineural invasion: East Asian retrospective series only, four different positivity thresholds, and a graded rather than binary outcome definition in Koide 2006.
	Imprecision	**Serious (−1)**	Serious (−1). Only 115 events across 351 patients. Three of the four individual estimates include unity (Koide 2006 0.90–11.40; Zhang 2014 0.24–3.87; Zhang 2021-R2 0.83–3.95), and the lower bound of the pooled interval (1.22) lies close to the null, so the evidence is compatible with an association that is only marginal.
	Publication bias	**Not serious**	Not serious, but suspected. As for perineural invasion.
	**Certainty**	**⊕○○○ Very low**	*Starting at high certainty for prognostic factor evidence, downgraded as set out above. No upgrade was applied, because residual concerns about bias and indirectness remain in every analysis.*
**Lymph node metastasis**	Study limitations	**Serious (−1)**	Serious (−1). Four cohorts rated at moderate to high overall risk of bias, none of which adjusted the association; two of the four contributing datasets come from a single institution and were scored on tissue microarrays, which may under-sample heterogeneous staining.
	Inconsistency	**Not serious**	Not serious. No statistical heterogeneity (τ^2 = 0.00; Chi² = 0.91, df = 3, p = 0.82; I^2 = 0%) and a consistent direction across all four cohorts.
	Indirectness	**Serious (−1)**	Serious (−1). East Asian retrospective series only; CD74 positivity defined by three different thresholds; nodal status classified under two different AJCC editions (7th in Zhang 2014 and Nagata 2009, 8th in Zhang 2021).
	Imprecision	**Serious (−1)**	Serious (−1). Although the pooled interval excludes unity, no individual cohort reached statistical significance and the optimal information size is not met for an odds ratio of this magnitude with 172 events; the interval (1.27–2.95) remains compatible with an association too small to be clinically useful.
	Publication bias	**Not serious**	Not serious, but suspected. As above.

Outcome	Domain	Judgement	Reason
	**Certainty**	**⊕○○○ Very low**	*Starting at high certainty for prognostic factor evidence, downgraded as set out above. No upgrade was applied, because residual concerns about bias and indirectness remain in every analysis.*
**Vascular permeation**	Study limitations	**Serious (−1)**	Serious (−1). Three cohorts, one at high overall risk of bias (Nagata 2009), none adjusted. Figure 5 as currently drawn carries the cohort labels for Nagata 2009 and Zhang 2014 in transposed order, which does not alter the pooled estimate but must be corrected.
	Inconsistency	**Not serious**	Not serious. No statistical heterogeneity ($\tau^2 = 0.00$; $Chi^2 = 0.54$, $df = 2$, $p = 0.76$; $I^2 = 0\%$).
	Indirectness	**Serious (−1)**	Serious (−1). East Asian retrospective series only; differing positivity thresholds; Koide 2006 again contributed a graded rather than binary contrast.
	Imprecision	**Very serious (−2)**	Very serious (−2). Only 46 events across 181 patients, and the confidence interval (0.83–4.68) is compatible both with no association and with a more than four-fold increase in odds. This is the least precise of the six analyses.
	Publication bias	**Not serious**	Not serious. The pooled result is null, so inflation by preferential publication of positive findings is not a plausible explanation for it.
	**Certainty**	**⊕○○○ Very low**	*Starting at high certainty for prognostic factor evidence, downgraded as set out above. No upgrade was applied, because residual concerns about bias and indirectness remain in every analysis.*
**Advanced pTNM stage**	Study limitations	**Serious (−1)**	Serious (−1). Three cohorts, one at high overall risk of bias, none adjusted; the counts extracted from Zhang 2014 for this endpoint (29 and 17) again do not match that cohort's group sizes (32 and 14).
	Inconsistency	**Not serious**	Not serious. No statistical heterogeneity ($\tau^2 = 0.00$; $Chi^2 = 0.20$, $df = 2$, $p = 0.90$; $I^2 = 0\%$), although the power of this test with three cohorts is minimal.
	Indirectness	**Very serious (−2)**	Very serious (−2). The three cohorts do not contribute the same contrast: Nagata 2009 compares stage III+IV with I+II, Zhang 2021-R2 compares stage III with I+II, and Zhang 2014, which by design enrolled only stage I–II patients, can contribute only stage II versus stage I. The pooled estimate therefore describes a general association with more advanced stage rather than any single, uniformly defined comparison. Stage was also classified under two different AJCC editions.
	Imprecision	**Serious (−1)**	Serious (−1). Only 94 events across 284 patients, and the confidence interval (0.93–2.82) includes unity.
	Publication bias	**Not serious**	Not serious. The pooled result is null.
	**Certainty**	**⊕○○○ Very low**	*Starting at high certainty for prognostic factor evidence, downgraded as set out above. No upgrade was applied, because residual concerns about bias and indirectness remain in every analysis.*
**Overall survival**	Study limitations	**Serious (−1)**	Serious (−1). Hong 2023 was rated at high overall risk of bias in the QUIPS assessment, principally because the survival cohort is a public transcriptomic dataset with no eligibility criteria, no baseline description and no reportable follow-up, and because the expression threshold is a software default; Nagata 2009 was also rated high, because its ≥70% threshold was selected on the basis of maximal prognostic separation with other thresholds examined but not reported. Against this, two of the three estimates are multivariable-adjusted, which is unusual in this literature and limits the downgrade to one level.
	Inconsistency	**Not serious**	Not serious. All three cohorts favour CD74-negative patients, there is no statistical heterogeneity ($\tau^2 = 0.00$; $Chi^2 = 1.47$, $df = 2$, $p = 0.48$; $I^2 = 0\%$), and the test for subgroup differences between the immunohistochemistry and transcript-level subgroups is not significant ($Chi^2 = 0.76$, $df = 1$, $p = 0.38$).
	Indirectness	**Serious (−1)**	Serious (−1). Two distinct sources. The prognostic factor is measured in two non-interchangeable ways, as tissue protein in two cohorts and as bulk-tissue transcript abundance in the third, and the two adjusted estimates were adjusted for different variable sets, neither of which included tumour grade or resection margin status, so the pooled hazard ratio is not an interpretable adjusted effect. All cohorts are again East Asian, and one contributed only after reconstruction of the hazard ratio from a published survival curve.
	Imprecision	**Not serious**	Not serious. The confidence interval (1.45–2.88) excludes unity, both bounds indicate a clinically important increase in hazard, and the estimate is essentially unchanged in the immunohistochemistry subgroup (2.21, 1.50–3.25).

Outcome	Domain	Judgement	Reason
	Publication bias	**Not serious**	Not serious, but suspected. Formal testing was not possible. We note that Zhang 2021 reports survival data for 170 further patients that are not included in this analysis; if that cohort is added in a future update the pooled estimate may change.
	**Certainty**	**⊕⊕○○ Low**	*Starting at high certainty for prognostic factor evidence, downgraded as set out above. No upgrade was applied, because residual concerns about bias and indirectness remain in every analysis.*
